# Cure of recurring *Klebsiella pneumoniae* carbapenemase-producing *Klebsiella pneumoniae* septic shock episodes due to complicated soft tissue infection using a ceftazidime and avibactam-based regimen: a case report

**DOI:** 10.1186/s13256-018-1934-2

**Published:** 2019-01-22

**Authors:** Giustino Parruti, Antonella Frattari, Ennio Polilli, Vincenzo Savini, Antonina Sciacca, Augusta Consorte, Donatella Concetta Cibelli, Adriana Agostinone, Francesco Di Masi, Alessandro Pieri, Pierluigi Cacciatore, Giancarlo Di Iorio, Paolo Fazii, Tullio Spina

**Affiliations:** 1 Infectious Diseases Unit, Pescara General Hospital, Via Fonte Romana 8, 65124 Pescara, Italy; 2 Anaesthesia and Intensive Care Unit, Pescara General Hospital, Pescara, Italy; 3 Clinical Pathology Unit, Pescara General Hospital, Pescara, Italy; 4 Microbiology and Virology Unit, Pescara General Hospital, Pescara, Italy

**Keywords:** Sepsis, *Klebsiella pneumoniae* carbapenemase (KPC)-producing *Klebsiella pneumoniae*, Treatment

## Abstract

**Background:**

Infections caused by multidrug-resistant Enterobacteriaceae are hard to treat and life-threatening due to reduced therapeutic options. Systemic infections caused by *Klebsiella pneumoniae* carbapenemase-producing *Klebsiella pneumoniae* strains have increased in many European regions, becoming frequent in many clinical settings, and are associated with high mortality. The co-formulation of ceftazidime, a third-generation cephalosporin, with avibactam, a new suicide inhibitor beta-lactamase inhibitor able to block most *Klebsiella pneumoniae* carbapenemases, has been recently licensed, with promising results in patients with limited or absent therapeutic options. Little is known, however, as to the efficacy of such a combination in patients with soft tissue infections caused by multidrug-resistant *Klebsiella pneumoniae* carbapenemase-producing strains of *Klebsiella pneumoniae*.

**Case presentation:**

A Caucasian 53-year-old man with paraplegia suffered multiple vertebral fractures due to a car crash. He was treated with external fixators that became infected early after insertion and were repeatedly and inefficiently treated with multiple antibiotics. He suffered repeated septic episodes caused by *Klebsiella pneumoniae* carbapenemase-producing *Klebsiella pneumoniae* strains with a multidrug-resistant profile. Meropenem, tigecycline, and colistin combinations allowed only temporary improvements, but septic shock episodes recurred, in spite of removal of infected external fixators. After approval of pre-marketing prescription by our local Ethics Committee, full clinical resolution was obtained with a compassionate treatment using meropenem and ceftazidime/avibactam in combination for 16 days.

**Conclusions:**

Our experience provides additional evidence that ceftazidime/avibactam, possibly in combination with meropenem rescued by avibactam, may be an efficacious treatment option also for complicated skin and soft tissue infections caused by multidrug-resistant strains of *Klebsiella pneumoniae* carbapenemase-producing *Klebsiella pneumoniae*.

## Introduction

Systemic infections caused by Gram-negative multidrug-resistant (MDR) Enterobacteriaceae are nowadays frequently hard to treat and life-threatening worldwide due to reduced therapeutic options [1]. *Klebsiella pneumoniae* carbapenemase (KPC)-producing *Klebsiella pneumoniae* (KPC-Kp) strains are endemic in most Italian regions and selection of nearly panresistant strains has become frequent in many clinical settings [2]. In particular, patients with difficult or delayed infectious source control may present with recurring infections and relapsing septic episodes, whose treatment may become increasingly difficult due to stepwise selection of bacterial strains with worsening resistance profiles. In such cases, effective source control may turn out to be useless even in immunocompetent patients, if clearance of residual infectious foci is impossible due to bacterial resistance [3]. In this scenario, availability of new therapeutic options may be crucial for patients’ rescue in the event of overwhelming septic recurrences. Recently, the US Food & Drug Administration/European Medicines Agency’s (FDA/EMA) release of a fixed dose combination of avibactam, a new carbapenemase inhibitor, and ceftazidime ushered expectation that at least some severe infections due to KPC-Kp may find a rescue option [4]. Experiences on the off-label use of such a combination for indications outside those included in clinical trials, however, are so far scanty. Here we present the case of an immunocompetent patient with vertebral traumatic fractures treated with multiple indwelling fixators, who became infected with a KPC-Kp strain early in the postoperative period. He could be rescued with the compassionate introduction of avibactam/ceftazidime as a last chance combination regimen after effective source control.

## Case presentation

**Fig. 1:**
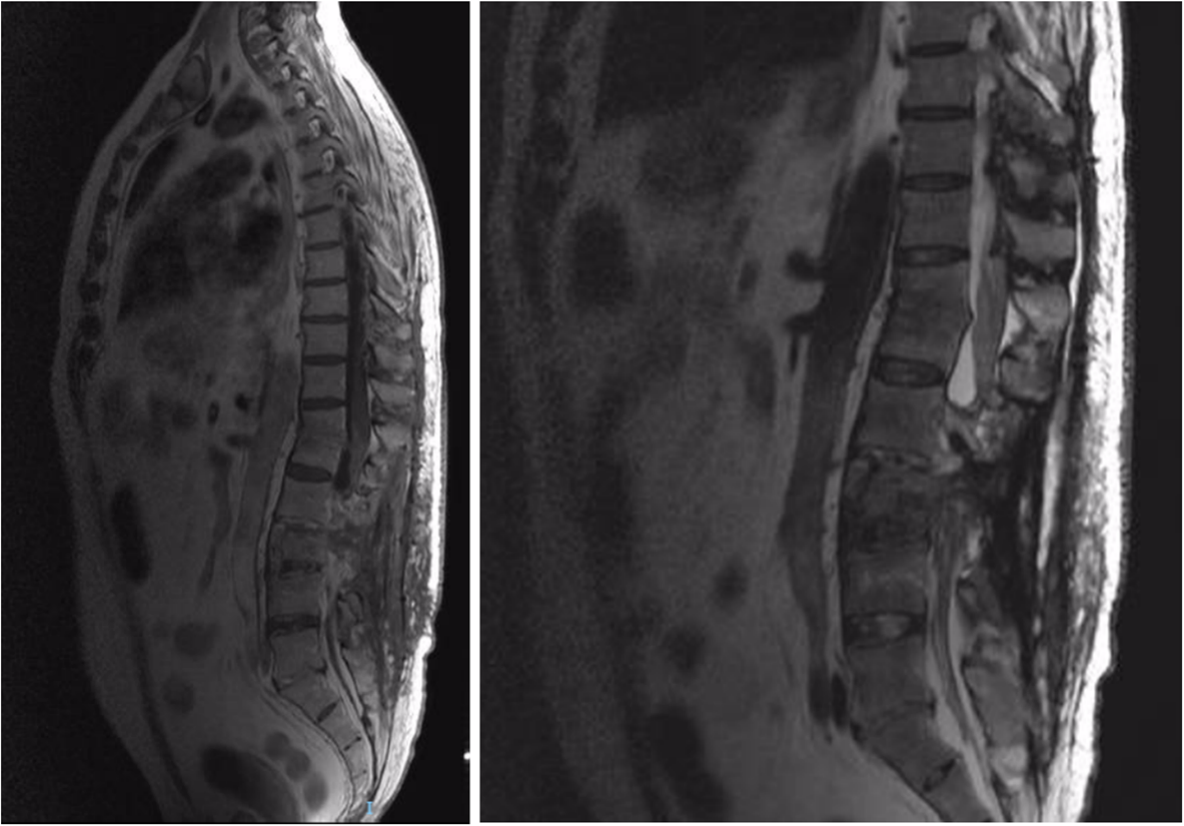
Vertebral and paravertebral infectious foci detected at vertebral magnetic resonance imaging after neurosurgical intervention for final removal of indwelling fixators

Our patient is a Caucasian 53-year-old, otherwise healthy, man with paraplegia since his recent car crash causing multiple vertebral fractures and a D7 lesion. He was admitted at the Infectious Diseases Unit due to low grade intermittent fever, severe back pain, and high (7.5 ng/mL) procalcitonin (PCT) levels in spite of the absence of any other sign of sepsis or septic shock. Repeated blood cultures (BCs), however, all turned positive for a single infecting strain of KPC-Kp (Table 1). Strains were molecularly typed as KPC II positive, with limited therapeutic options (Table 2). He was treated with meropenem, tigecycline, and colistin, in accordance with local protocols for KPC-Kp (Table 2). At that time, he refused any surgical management as he had been treated at another Italian center for his previous three septic episodes following insertion of fixators. In those circumstances, due to recent vertebral stabilization, he had been treated with single shot removal and replacement surgery for infected fixators, followed by early relapse of infection signs. After 18 days of treatment in our ward, the infection apparently resolved, with negative control BCs, and normal C-reactive protein (CRP) and PCT levels. He was discharged to home, with the indication to monitor infection relapse twice weekly, while starting his rehabilitation protocol. After 2 weeks, with normal PCT levels, his CRP had risen to 79 mg/L. He complained of worsening back pain. After 10 days, he was re-hospitalized on emergency due to recurrent sepsis. His BCs were again positive for KPC-Kp (Table 1). Treatment was restarted with the same combination based on the available resistance profile (Table 2). Treatment was again efficacious, and on the 12th day he accepted his transfer to the Neurosurgery Unit for removal of fixators (Fig. 1). It was explained to him that control neuroimaging studies allowed a two-step procedure, aimed at a definitive cure of infection prior to possible reinsertion of fixators. He was given the same antibiotic treatment for residual source control after surgery for 21 days (Table 2). He was once more discharged to home as neurosurgeons considered reinsertion of fixators unnecessary. After 35 days, he was readmitted with recurring sepsis. BCs revealed progression of the resistance phenotype of his KPC-Kp isolates (Table 1). A rescue treatment was provided with all available, potentially useful antibiotics, including gentamycin and colistin (Table 2). Clinical remission was obtained after 14 days of treatment, but septic shock recurred 6 days after treatment discontinuation. He presented with a relapse of hyperpyrexia (42 °C), hypotension, severe leukocytosis with white blood cells (WBC) 38,000, drop in platelet counts (nadir 46,000/mm^3^), and rapidly ensuing renal failure with creatinine nadir of 4.4 mg/dL and liver failure with alanine aminotransferase (ALT) nadir of 456 U/L.

**Table 1:** Evolving phenotypes of isolated *Klebsiella* strains from our patient

Antibiotics	April 10, 2016	July 23, 2016	December 27, 2016		January 19, 2017
	*Klebsiella pneumoniae*	*Klebsiella pneumoniae*	*Klebsiella pneumoniae*	*Enterobacter aerogenes*	*Klebsiella pneumoniae*
Amikacin	Resistant	Resistant	Resistant	Susceptible	Resistant
Amoxicillin-clavulanic acid	Resistant	Resistant	Resistant	Resistant	Resistant
Ampicillin	Resistant	Resistant	Resistant	Resistant	Resistant
Cefepime	Resistant	Resistant	Resistant	Susceptible	Resistant
Cefotaxime	Resistant	Resistant	Resistant	Susceptible	Resistant
Ceftazidime	Resistant	Resistant	Resistant	Susceptible	Resistant
Ciprofloxacin	Resistant	Resistant	Resistant	Susceptible	Resistant
Colistin	Susceptible	Susceptible	Resistant	Susceptible	Resistant
Ertapenem	Resistant	Resistant	Resistant	Susceptible	Resistant
Fosfomycin	Susceptible	Susceptible	Resistant	Susceptible	Resistant
Gentamicin	Intermediate	Intermediate	Intermediate	Susceptible	Intermediate
Imipenem	Resistant	Resistant	Resistant	Susceptible	Resistant
Meropenem	Resistant	Resistant	Resistant	Susceptible	Resistant
Piperacillin/tazobactam	Resistant	Resistant	Resistant	Susceptible	Resistant
Tigecycline	Intermediate	Intermediate	Resistant	Susceptible	Intermediate
Trimethoprim/sulfamethoxazole	Resistant	Resistant	Resistant	Susceptible	Resistant

**Table 2:** Combination regimens used to treat ensuing septic episodes in our patient

*Date of hospitalization in Infectious Diseases Unit*	Microbiological data	Combination therapy	Doses	Timing of therapy
*10 April 2016* *(first septic episode)*	KPC-producing *Klebsiella pneumoniae* isolates on BCs	Tigecycline Meropenem Colistin	LD 100 mg + 50 mg/12 hours LD 2gr + 2gr/8 hours LD 9 MU + 4.5 MU/8 hours	18 days
*June 2016* *Re-hospitalization for the second septic episode*	KPC-producing *Klebsiella pneumoniae* on BCs	Tigecycline Meropenem Colistin	LD 100 mg + 50 mg/12 hours LD 2gr + 2gr/8 hours LD 9 MU + 4.5 MU/12 hours	12 days before surgical management for fixators’ removal
*Continuing hospitalization after surgical treatment*		Same combination therapy as above		21 days
*December. 2016* *Third septic recurrence with hospitalization*	KPC-producing *Klebsiella pneumoniae* and *Enterobacter aerogenes* isolates on BC	Tigecycline Meropenem Colistin Gentamycin	LD 100 mg + 50 mg/12 hours LD 2gr + 2gr/8 hours LD 9 MU + 4.5 MU/8 hours	14 days
*Septic shock/MOF after discontinuation of therapy* *(January 2017)*	KPC-producing *Klebsiella pneumoniae* with a progression of a resistance phenotype isolates on BC	Avibactam/ceftazidime (compassionate use) Tigecycline Meropenem Gentamycin	2gr/8 hours LD 100 mg + 50 mg/12 hours LD 2gr + 2gr/8 hours LD 7 mg/kg per day (480 mg) + 5 mg/kg per day (350 mg/24 hours)	16 days

*BCs* blood cultures, *KPC Klebsiella pneumoniae* carbapenemase, *LD* loading dose, *MOF* multiple organ failure, *MU* monitor unit

During his last hospital stay, our patient was positive for other microbiological culture assays: susceptible *Candida albicans* from an epicutaneous catheter and BCs; susceptible *Pseudomonas aeruginosa* from urine cultures, and susceptible *Staphylococcus hominis* from BCs. All viral tests performed were negative: cytomegalovirus **(**CMV) DNA in whole blood samples; Epstein–Barr virus **(**EBV) DNA in whole blood samples; serum hepatitis B surface antigen **(**HBsAg), and hepatitis C virus **(**HCV) antibodies, as recommended by national and local guidelines for opt out tests before surgical interventions. Respiratory pathogens were not tested in the absence of clinical or radiological suspicion of lung involvement at any time during his clinical course. HCV antibodies and the HBsAg were assayed with chemiluminescent immunoassays; antibiotic susceptibility was assayed with Vitek® (bioMérieux). An Xpert® Carba-R (Cepheid; Sunnyvale, USA) was used for rapid detection of resistance to carbapenems. Molecular tests for EBV and CMV were performed with ELITe MGB® Kits for real-time polymerase chain reaction (PCR) kits (Turin, Italy). Altered mentation and clonal involuntary movements at upper limbs completed the presenting picture. Lactates from arterial blood rose to 4 mmol. Septic shock support was provided, including fluid resuscitation, noradrenaline, and albumin support. The best possible antibiotic rescue regimen, that is the same regimen as 20 days earlier, was established in accordance with available microbiological data (Tables 1 and 2). Meanwhile, compassionate use of avibactam/ceftazidime was requested and authorized on emergency by the local Ethical Board. A new treatment regimen was started on day 5, including avibactam/ceftazidime, meropenem, gentamycin, and tigecycline. This treatment provided rapid and stable control of sepsis, with normal inflammation indexes on day 10. The regimen was continued for 16 days. Microbiological evidence of *in vitro* efficacy of the combination of ceftazidime, avibactam, and meropenem was provided. Our patient was discharged on day 31 to a local rehabilitation facility, with remarkable improvements. He was finally discharged home after 56 days. His PCT, CRP, and control BCs were all negative 40 days after discharge. His last follow-up visit was on December 5, 2017, with persistently normal clinical and laboratory parameters.

## Discussion and conclusion

Our patient was a previously healthy man, surviving a car crash with paraparesis due to a D7 spinal lesion. Due to multiple vertebral fractures, multiple fixators were positioned along his spine, with ensuing early infective complications. His skin and soft tissue infections never resolved until definitive removal of fixators, nor were they resolved when residual infection was repeatedly treated with suboptimal regimens for his deep-seated KPC-Kp infection. In fact, combination antibiotic regimens including gentamycin, meropenem, tigecycline, and colistin, all delivered at the recommended doses and for expanded periods of time as recommended in the recent international literature for such critical infections [5], were only able to provide short spells of remission, with apparent remission of sepsis indexes. His last relapsing infection episode, in particular, was a frank septic shock with reversible multiorgan failure, prompting us to plead for compassionate use of avibactam/ceftazidime. Recent literature data suggested that avibactam may be efficacious on most strains of KPC-Kp [6], and this looked appealing in our case, although his infection site was not included in available clinical trials. As avibactam-containing combinations were experimented on a limited subset of possible clinical conditions, including nosocomial pneumonia, abdominal infections, and complicated urinary tract infections [4, 7, 8], little is known at present on the potential benefit that complicated and long-lasting soft tissue infections, with a possible bone involvement as in the case of our patient, may draw from introduction of avibactam/ceftazidime or avibactam/ceftazidime including rescue antibiotic combinations. Meropenem has a good soft tissue penetration [9], and it provided repeated transient benefit for this patient when included in previous regimens at high dose and protracted infusion times. Avibactam provided in the fixed dose combination with ceftazidime was likely to potentiate meropenem efficacy in our case. Microbiological evidence of avibactam efficacy could be obtained on recent isolates from our patient. Although direct evidence for *in vitro* enhanced combination efficacy could not be obtained, it is likely that the infusion of meropenem for expanded times in sequence with avibactam/ceftazidime may have greatly helped to reach the target of clinical eradication. Other susceptible pathogens evidenced by microbiological assays were easily controlled by standard prescribed treatments.

As to the duration of treatment, we doubted whether a second shipment of the drug should be requested in view of clinical success. Most literature evidence would suggest that longer duration of treatment beyond 2 weeks in the absence of signs of residual infection would not be of benefit [10]. In fact, control MR imaging showed remarkable improvement of lesions, in the absence of the need for further infection control surgery.

Our experience therefore provides a useful piece of evidence that avibactam-containing regimens may support treatment of KPC-Kp susceptible strains also in extreme circumstances, such as recurrent sepsis and septic shock episodes from soft tissue infections with delayed surgical source control.

## Abbreviations

BC: Blood culture
CMV: Cytomegalovirus
CRP: C-reactive protein
EBV: Epstein–Barr virus
HBsAg: Hepatitis B surface antigen
HCV: Hepatitis C virus
KPC: *Klebsiella pneumoniae* carbapenemase
KPC-Kp: *Klebsiella pneumoniae* carbapenemase-producing *Klebsiella pneumoniae*
PCT: Procalcitonin
